# NDUFS3 knockout cancer cells and molecular docking reveal specificity and mode of action of anti-cancer respiratory complex I inhibitors

**DOI:** 10.1098/rsob.220198

**Published:** 2022-11-09

**Authors:** Ivana Kurelac, Beatrice Cavina, Manuela Sollazzo, Stefano Miglietta, Agnese Fornasa, Monica De Luise, Maria Iorio, Eleonora Lama, Daniele Traversa, Hamid Razi Nasiri, Anna Ghelli, Francesco Musiani, Anna Maria Porcelli, Luisa Iommarini, Giuseppe Gasparre

**Affiliations:** ^1^ Department of Medical and Surgical Sciences (DIMEC), University of Bologna, Bologna, Italy; ^2^ Centre for Applied Biomedical Research (CRBA), University of Bologna, Bologna, Italy; ^3^ Department of Pharmacy and Biotechnology (FABIT), University of Bologna, Bologna, Italy; ^4^ Interdepartmental Centre for Industrial Research ‘Scienze della Vita e Tecnologie per la Salute’, University of Bologna, Bologna, Italy; ^5^ Department of Cellular Microbiology, University Hohenheim, Stuttgart, Germany

**Keywords:** respiratory complex I, metformin, BAY 87-2243, EVP 4593, IACS-010759, complex I inhibitors, cancer therapy

## Abstract

Inhibition of respiratory complex I (CI) is becoming a promising anti-cancer strategy, encouraging the design and the use of inhibitors, whose mechanism of action, efficacy and specificity remain elusive. As CI is a central player of cellular bioenergetics, a finely tuned dosing of targeting drugs is required to avoid side effects. We compared the specificity and mode of action of CI inhibitors metformin, BAY 87-2243 and EVP 4593 using cancer cell models devoid of CI. Here we show that both BAY 87-2243 and EVP 4593 were selective, while the antiproliferative effects of metformin were considerably independent from CI inhibition. Molecular docking predictions indicated that the high efficiency of BAY 87-2243 and EVP 4593 may derive from the tight network of bonds in the quinone binding pocket, although in different sites. Most of the amino acids involved in such interactions are conserved across species and only rarely found mutated in human. Our data make a case for caution when referring to metformin as a CI-targeting compound, and highlight the need for dosage optimization and careful evaluation of molecular interactions between inhibitors and the holoenzyme.

## Graphical abstract

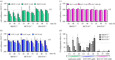

## 1. Introduction

Respiratory complex I (NADH:ubiquinone oxidoreductase; EC 1.6.5.3; CI) is a ubiquitous enzyme that constitutes the entry site of electrons coming from NADH in the mitochondrial respiratory chain. This process is coupled with proton translocation, thus contributing to the generation of the electrochemical gradient necessary for mitochondrial ATP production [1]. Owing to its pivotal contribution in controlling cell metabolism, adaptation to hypoxia and susceptibility to apoptosis, CI has been recognized as an essential player during tumour progression, metastasis formation and resistance to chemotherapy (reviewed in [2]). In particular, its derangement or inhibition has shown to slow down tumour growth, confining aggressive tumours into a low proliferative state and providing a temporal window for additional therapeutic intervention [3–7]. Hence, this enzyme has emerged as an attractive druggable target for cancer treatment, and several CI inhibitors have been proposed as possible therapeutic agents in different experimental preclinical settings [2,5,8–11]. Within the plethora of CI inhibitors, the biguanide metformin is the most widely investigated, being already used in clinical practice in the treatment of type 2 diabetes (T2D). Several *in vitro* and *in vivo* studies highlight the action of this drug in preventing tumour progression [4,12–15], while epidemiological data on T2D patients and observational studies link the use of metformin with lower incidence of neoplastic disease (reviewed in [16–18]). However, meta-analyses of clinical data revealed a heterogeneous patient response to metformin, depending on tumor type and stage [19,20] and conflicting results emerge also from completed clinical trials [21–30]. Further, recent studies showed CI-independent antiproliferative effects of metformin [31,32] and identified additional mitochondrial targets [32–35], posing the need for optimization of the biguanide doses for each tumour type and prompting the development of more specific compounds. Among the large group of repurposed or newly discovered molecules, BAY 87-2243 has shown to arrest cell proliferation in non-small cell lung cancer cells [36], to induce cell death in melanoma cells [37,38], thus preventing tumour growth *in vivo* [36–38] and to increase sensitivity to radiotherapy in squamous cell carcinoma [39]. Lastly, quinazoline-based compounds such as EVP 4593, originally identified as Nuclear Factor kappa-light-chain-enhancer of activated B cells (NF-κB) inhibitor, have been recently recognized to target specifically CI [40] and to strongly suppress tumour growth in colon cancer xenografts in association with vascular endothelial growth factor (VEGF) inhibition [41].

Despite the demonstrated antitumorigenic properties of these molecules and the advances in the development of new CI inhibitors, none of them is currently used in clinical practice and several open issues remain. First, the studies conducted so far did not consider the possible effects of the compounds on molecular targets different from CI, which poses the question of their specificity. Second, the inhibitors efficacy as antineoplastic agents has been determined in a limited number of cancer models, neglecting that it might depend on the tumour type and its metabolic status. Lastly, the mechanisms behind the effects of these molecules have rarely been dissected, most often associating their antiproliferative effect to apoptotic cell death [42,43]. In this frame, the recent release of high-resolution structures of mammalian CI in different conformations [44] encourages the investigation of the interactions between inhibitors and the enzyme at the atomic level, allowing to gain insights on their specificity and mode of action in a fast and cost-effective manner.

We here evaluated the antiproliferative potential and the effective concentrations of three CI inhibitors, namely metformin, EVP 4593 and BAY 87-2243, in cell lines representative of aggressive ovarian cancer, colon cancer and melanoma. By exploiting unique CI deficient cell models, we demonstrated a highly non-specific effect of metformin uncommon to the other inhibitors. Molecular docking analyses revealed that EVP 4593 and BAY 87-2243 form a tight network of interactions in the quinone binding site, although involving different residues, possibly explaining the difference in efficiency between the two inhibitors.

## 2. Material and methods

### 2.1. Cell lines generation, maintenance and treatments

Human ovarian cancer cell line SKOV3 and murine melanoma cell line B16-F10 were purchased from ATCC (#HTB-77; #CRL-6475, Manassas, VA, USA). Human colorectal cancer cell line HCT116 was authenticated using AMPFISTRIdentifiler kit (Applied Biosystem #4322288) and its STR profile corresponded to the putative background. Mitochondrial DNA (mtDNA) was sequenced by Sanger sequencing as previously described [45], to ensure *wild-type* genotype. The results are available upon request. NDUFS3 knockout cells, referred as SKOV3^−/−^, HCT116^−/−^ and B16-F10^−/−^, were generated by genome editing as previously reported [7,45,46]. Briefly, CRISPR/Cas9 system was used to introduce a frameshift mutation in *NDUFS3* gene in SKOV3 human cell line and *Ndufs3* gene in B16-F10 murine cell line, using exon 2 targeting guide TGTCAGACCACGGAATGATG and exon 3 targeting guide TTGTGGGTCACATCACTCCG, respectively. Instead, zinc finger endonuclease system was used to obtain HCT116 *NDUFS3* knockout. Plasmids containing cDNA of *NDUFS3-*targeted zinc finger endonucleases (Sigma-Aldrich #CKOZFND15186) were purified and transcribed *in vitro*. The pool of zinc finger endonucleases mRNAs was transfected into 70% confluent HCT116 cells. The resulting heterozygous clones were subjected to a subsequent second transfection with *NDUFS3* zinc finger pool and led to the selection of homozygous frameshift *NDUFS3* mutants [7].

Cells were grown in high-glucose Dulbecco's Modified Eagle's Medium (DMEM) plus sodium pyruvate (Euroclone #ECB7501L), supplemented with 10% FBS South America origin EU Approved (Euroclone #ECS5000L), 2 mM l-glutamine (Euroclone #ECB3000D), 1% penicillin/streptomycin (Euroclone #ECB3001D) and 50 µg ml^−1^ uridine (Sigma-Aldrich #U3003), in an incubator at 37°C with a humidified atmosphere at 5% CO_2_. EVOS M5000 Imaging System (ThermoFisher Scientific #AMF5000) was used for cell lines monitoring.

### 2.2. Compounds

We tested the following CI inhibitors:1,1-dimethylbiguanide hydrochloride (metformin, Sigma-Aldrich #D150959), N4-[2-(4-phenoxyphenyl) ethyl]-4,6-quinazolinediamine (EVP 4593, Sigma-Aldrich #SML0579), 1-cyclopropyl-4-(4-[(5-methyl-3-{3-[4-(trifluoromethoxy) phenyl]-1,2,4-oxadiazol-5-yl)-1H-pyrazol-1-yl)methyl]pyridin- 2-yl) piperazine (BAY 87-2243, Sigma-Aldrich #SML2384) and IACS-010759 (SelleckChem #S8731). Metformin was directly dissolved at the indicated concentration in growth medium. EVP 4593, BAY 87-2243 and IACS-010759 were dissolved in dimethyl sulfoxide (DMSO, Sigma-Aldrich #D2650) to generate a 42 mM, 3.8 mM and 10 mM stock solution, respectively, that were stored at −20°C. Prior to each experiment, all the three drugs were freshly dissolved in the medium to reach the indicated final concentration. For each treatment, the final concentration of DMSO in the medium was calculated (less than or equal to 0.1%) and was used as vehicle control in the untreated condition (referred as UT).

### 2.3. Cell growth and morphology evaluation

Cell proliferation and morphology were assessed using the Incucyte Live-imaging System (Sartorius) and proliferation was quantified through time-lapse image acquisition and metrics application.

**Figure 1.**
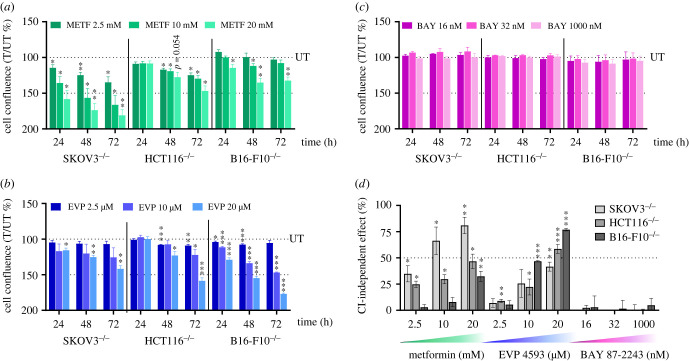
High concentrations of metformin and EVP 4593 reduce proliferation of cancer cells lacking CI. (*a*–*c*) Proliferation of SKOV3^−/−^, HCT116^−/−^ and B16-F10^−/−^ cells upon treatment with different concentrations of metformin (METF) (*a*), EVP 4593 (EVP) (*b*) and BAY 87-2243 (BAY) (*c*). Data are shown as cell confluence ratio of treated (*T*) to untreated (*UT*) control cells after 24, 48 and 72 h of incubation with the indicated compound concentrations. Cell confluence was determined by using the Incucyte Live-imaging System and at each time point was normalized on time 0 (*T*0). Data are mean ± s.e.m. (*n* ≥ 3) and one-sample *t-*test was performed for each treatment using 100 as reference value. Statistical significance is specified with asterisks (*p ≤ 0.05, **p ≤ 0.01, ***p ≤ 0.001). (*d*) CI-independent antiproliferative effect for each compound concentration in SKOV3^−/−^, HCT116^−/−^ and B16-F10^−/−^ cell lines at 72 h of inhibitor treatment. Data (mean ± s.e.m., *n* ≥ 3) are expressed as [100% (UT) – cell confluence ratio (T/UT %)] and one-sample *t-*test was performed for each treatment using 0 as reference value. Statistical significance is specified with asterisks (*p ≤ 0.05, **p ≤ 0.01, ***p ≤ 0.001).

**Figure 2.**
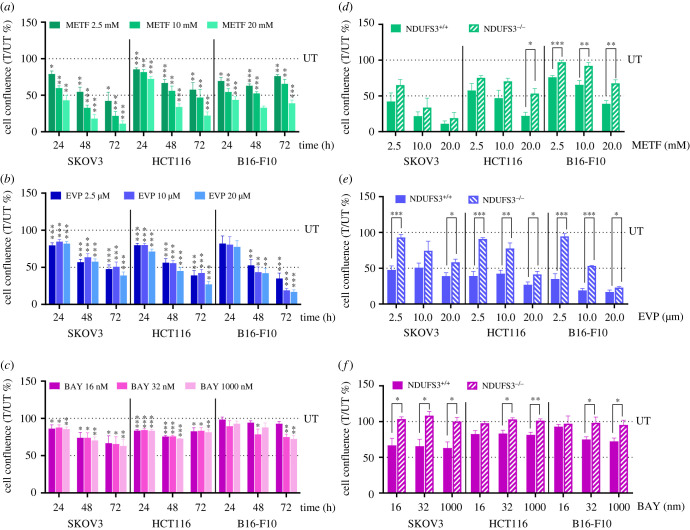
Proliferation of CI-competent cancer cells is slowed down by treatment with inhibitors. (*a*–*c*) Proliferation of SKOV3, HCT116 and B16-F10 cells upon treatment with different concentrations of metformin (METF) (*a*), EVP 4593 (EVP) (*b*) and BAY 87-2243 (BAY) (*c*). Data are expressed as cell confluence ratio of treated (*T*) to untreated (*UT*) control cells after 24, 48 and 72 h of incubation with the indicated compound concentrations in Incucyte Live-imaging System. Cell confluence at each time point was previously normalized on time 0 (T0). Data are mean ± s.e.m. (*n* ≥ 4) and one-sample *t-*test was performed using 100 as reference value. Statistical significance is specified with asterisks (*p ≤ 0.05, **p ≤ 0.01, ***p ≤ 0.001). (*d*,*e*) Comparison of cell proliferation of NDUFS3^+/+^ and NDUFS3^−/−^ cells treated with different concentrations of metformin (METF) (*d*), EVP 4593 (EVP) (*e*) and BAY 87-2243 (BAY) (*f*). Data are presented as cell confluence ratio of treated (*T*) to untreated (*UT*) control cells after 72 h of incubation with the indicated compounds concentrations; cell confluence was previously normalized on time 0 (*T*0). Data are mean ± s.e.m. (*n* ≥ 4) and Student *t-*test was performed between NDUFS3^+/+^ and NDUFS3^−/−^ cells, for each cell line and for each treatment condition. Statistical significance is specified with asterisks (*p ≤ 0.05, **p ≤ 0.01, ***p ≤ 0.001).

Briefly, 2.5 × 10^3^ SKOV3/well, 2 × 10^3^ HCT116/well and 1 × 10^3^ B16-F10/well were seeded in 96-well plates in high-glucose DMEM and incubated overnight to allow cells attachment. For specificity assays, NDUFS3 *wild-type* and knockout cells were seeded simultaneously, and the same confluence mask was applied for subsequent analysis. Treatment with CI inhibitors (metformin, EVP 4593, IACS-010759 and BAY 87-2243) started 24 h after seeding, and the plate was placed in Incucyte for 72 h at 37°C with a humidified atmosphere at 5% CO_2_. Images (4/well) were captured at 10× magnification with 2 h intervals. Cell growth was evaluated by Incucyte system phase contrast software (confluence metrics), which provides an occupied area average (% of confluence) per image/per well. For data in electronic supplementary material, figures S1 and S2, the analysis was performed using % of confluence metric per scan normalized on treatment starting point (time 0). For data in figures 1 and 2, the percentage of cell confluence normalized on time 0 for each inhibitor's treatment was subsequently normalized to untreated control. At least three replicate experiments were performed for each treatment condition.

### 2.4. Apoptotic cell death evaluation

Apoptosis upon drug treatments was determined using the Incucyte Caspase-3/7 green dye (Sartorius #4440). Cells (SKOV3, HCT116 and B16-F10) were seeded in a 96-wells plate in high glucose DMEM, as described above. Drugs were added after 24 h, in presence of 5 µM Incucyte Caspase-3/7 green dye following the manufacturer's protocol. Then, the plate was placed in Incucyte for 72 h and images (4/well) were captured at 10× magnification with 2 h intervals, both in phase contrast and fluorescent green channel. For each cell line, staurosporine (100 nM, Sigma-Aldrich #S4400) treatment was performed as a positive control. For each experimental condition, the masks for fluorescent objects identification were set by the Incucyte integrated analysis software. Total and green positive cells were counted at 10× magnification in three fields of view per condition, and data are expressed as percentage of apoptotic cells [(number of green cells/number of total cells) × 100].

### 2.5. Microrespirometry

Mitochondrial respiration was measured using the protocol described for the Seahorse XFe Cell Mito Stress Test Kit (Agilent #103015-100) following the manufacturer instructions. Briefly, 12 × 10^3^ cells/well (SKOV3, HCT116) and 8 × 10^3^ (B16-F10) were seeded in 80 µl of DMEM medium into XFe96 cell culture plates and incubated for 48 h (HCT116) and for 24 h (SKOV3, B16-F10) at 37°C and 5% CO_2_. Complete growth medium was replaced with 180 µl of XF medium (Agilent #103575-100) supplemented with 10 mM glucose, 1 mM sodium pyruvate, 2 mM l-glutamine, at pH 7.4. For temperature and pH equilibration, cells were incubated at 37°C for 30 min. Three oxygen consumption rate (OCR) baseline measurements were followed by injection of either vehicle or different concentrations of CI-inhibitors and subsequent measurements; 1 µM oligomycin, carbonyl cyanide-*p*-trifluoromethoxyphenylhydrazone (FCCP), 1 µM rotenone and 1 µM antimycin A were sequentially added to each well. FCCP concentrations were previously optimized by titration and were 0.5 µM for SKOV3, 0.75 µM for HCT116 and 1 µM for B16-F10, respectively. At the end of the assay, medium was removed and sulforhodamine B (SRB) assay was performed to determine protein content for normalization. Briefly, plates were incubated with 10% trichloroacetic acid (TCA) for 1 h at 4°C to fix the cells. Five washes in water were carried out. Once the plates were dry, proteins were stained by incubation with 0.4% SRB for 30 min at RT. SRB was solubilized with 10 mM Tris and absorbance at 560 nm was determined using a Victor2 plate reader (Perkin-Elmer). Data (pmol min^−1^) were normalized to blank corrected SRB absorbance.

### 2.6. Mitochondrial ATP synthesis rate assay

The rate of mitochondrial ATP synthesis was measured in digitonin-permeabilized CI competent cells by using a luciferin/luciferase assay as previously described with minor modifications [47]. Chemiluminescence was determined as a function of time with a luminometer (Sirius L Tube, Titertek-Berthold, Pforzheim, Germany). After trypsinization, cells (10 × 10^6^ ml^−1^) were suspended in a buffer containing 150 mM KCl, 25 mM Tris–HCl, 2 mM EDTA (ethylenediaminetetraacetic acid), 0.1% bovine serum albumin, 10 mM potassium phosphate, 0.1 mM MgCl_2_, pH 7.4, kept at room temperature for 15 min, then incubated with 50 µg ml^−1^ digitonin until 90–100% of cells were positive to Trypan Blue staining. Aliquots of 3 × 10^5^ permeabilized cells were incubated in the same buffer in the presence of the adenylate kinase inhibitor P1,P5-di(adenosine-5′) pentaphosphate (0.1 mM, Sigma-Aldrich #A2754) and 10 µl of the luciferin/luciferase assay reagent (Sigma-Aldrich #FLAAM-5VL). Then, ATP synthesis rate was determined after the sequential addition of the following substrates and inhibitors. First, 0.9 mM sodium malate (Sigma-Aldrich #M1125), 0.9 mM sodium pyruvate (Sigma-Aldrich #P2256) and 0.09 mM ADP (Sigma-Aldrich #A2754) were added to determine ATP synthesis via CI for at least 30 s after injections. Then, CI inhibitors were added at the optimized concentrations that completely suppress maximal respiration previously determined by titration with Seahorse. Subsequently, 2 µg ml^−1^ rotenone and 10 mM succinate were provided to determine CII-driven ATP synthesis without any interference of possible residual activity derived from CI. The chemiluminescence signal was calibrated with an internal ATP standard after the addition of 1 µM oligomycin. The rates of the ATP synthesis were normalized to protein content and the citrate synthase (CS) activity.

### 2.7. Molecular docking

Among the recently released high-resolution CI ovine structures [44] we focused on three of them: one for the initial step of the catalytic cycle (PDB ID 6ZKI), in which a molecule of decyl-ubiquinone is indeed present in the shallow quinone binding site (Q-site), the second in the close conformation corresponding to the quinone reduction step (PDB ID 6ZKC), in which a molecule of decyl-ubiquinone was solved in both the shallow and the deep Q-sites, and one of the rotenone inhibited structures (PDB ID 6ZKK) in which a single rotenone molecule is found in the deep Q-site. Docking calculations were conducted in two stages: in the first benchmark redocking stage the ligand (i.e. rotenone or decyl-ubiquinone) present in some selected structures of ovine CI were removed and the ability of the selected protocol to restore the proper binding pose was tested. In the second stage, EVP 4593 and BAY 87-2243 were docked to the Q-site with the aim of obtaining new information regarding the structure–function relationship of these inhibitors and their different behaviour. CI structures used for the docking calculations where treated as follows: hydrogen atoms were added by using the *addh* tool available in the UCSF Chimera suite [48], the atomic charges were assigned by using the AMBER ff14SB force field [49], and the proteins were oriented so that they could contain the Q site within a parallelepiped box of the smallest possible size. The charges for the [Fe4S4] clusters were assigned according to Na *et al*. [50]. The ligand charges were assigned by using the AM1-BCC method [51]. Ligands that were not present in the selected structures were generated with UCSF Chimera and minimized through 1000 step of steepest descent and 100 steps of conjugate gradient using the same software. The analysis of the Q site channel was performed by using the MOLEonline web server [52,53]. The docking calculations were performed by using AutoDock Vina 1.0 [54] with default parameters. The analysis of the results was made by using UCSF Chimera and LigPlot [55].

### 2.8. Multiple sequence alignment

Multiple sequence alignment was performed as previously reported [56]. Briefly, all of the reviewed non-redundant eukaryotic protein sequences from ND1, NDUFS2 (49 kDa in ovine CI) and NDUFS7 (PSST in ovine CI) were downloaded from UniProtKB (https://www.uniprot.org/help/uniprotkb, accessed on 15 May 2022). Ovine 49 kDa and PSST sequences were obtained by 6ZKC, 6ZKI and 6ZKK structures used for molecular docking analyses. Fragment sequences were eliminated obtaining a final number of aligned sequences of 159 for ND1, 24 for NDUFS2 and 18 for NDUFS7. Sequences were aligned using Clustal Omega v:1.2.4 (https://www.ebi.ac.uk/Tools/msa/clustalo/, accessed on 15th May 2022) with the default parameters. Numbering of nuclear subunits does not consider the mitochondrial translocation sequence (MTS). Ovine sequences were set as reference for numbering and consensus sequences and percentages of identity were calculated for each alignment by using Jalview v:2.11.1.4 [57]. Amino acids were considered conserved if their sequence identity surpassed the conservation threshold of 70%. Data are reported in electronic supplementary material, table S1.

### 2.9. Genetic variations search

In order to identify variants in *MT-ND1*, *NDUFS2* and *NDUFS7* potentially affecting EVP 4593 and BAY 87-2243 interactions in the Q-site, we exploited Ensembl data sources (GRCh38.p13; Ensembl IDs: MT-ND1—ENSG00000198888; NDUFS2—ENST00000367993.7; NDUFS7—ENST00000233627.14). In particular, dbSNP (https://www.ncbi.nlm.nih.gov/snp/) was interrogated to find already reported variations, while ClinVar (https://www.ncbi.nlm.nih.gov/clinvar/) was used for variants of clinical significance. Somatic variants within the Q-site already associated with cancer were annotated *via* COSMIC (https://cancer.sanger.ac.uk/cosmic) search. When available, the minor allele frequency (MAF) relative to each variant was determined by frequencies reported in dbSNP, exploiting pipelines and databases containing exomes and genomic sequences such as ALFA, 8.3KJPN, KOREAN population from KRGDB, HGDP, MGP and others. Also, GnomAD/ExAC database (https://gnomad.broadinstitute.org/), that contains large-scale sequencing data obtained from specific diseases and population genetic studies, was consulted to interpret human genetic variations. Frequencies of mtDNA variants are referred to the revised Cambridge Reference Sequence (rCRS, Accession Number NC 012920.1) and were reported according to the human mtDNA variation databases Mitomap (https://www.mitomap.org/MITOMAP) and Helix (https://www.helix.com/pages/mitochondrial-variant-database). Non-synonymous variants and their relative frequencies are reported in tables 1 and 2.

**Table 1.** Nuclear genome variants and amino acid position within the mature protein, following mitochondrial targeting sequence cleavage (-MTS) and within the preprotein, including the mitochondrial targeting sequence (+MTS); (*) asterisk indicates the translational stop codon; (-) dash indicates frameshift variants. ExAC: Exome Aggregation Consortium (https://exac.broadinstitute.org/); GnomAD: Genome Aggregation Database (https://gnomad.broadinstitute.org/); TOPMed: Trans-Omics for Precision Medicine (https://topmed.nhlbi.nih.gov/); ALFA: Allele Frequency Aggregator (https://www.ncbi.nlm.nih.gov/snp/docs/gsr/alfa/); ALSPAC: Avon Longitudinal Study of Parents and Children (http://www.bristol.ac.uk/alspac/); TWINSUK: Twins UK (https://twinsuk.ac.uk/).

Nucleotide change	Aa change (−MTS/+MTS)	MAF % (*n*. variants/total samples)	Source(s)	Notes
***NDUFS2***				
c.278G > A	p.G60D/p.G93D	not available	COSMIC: COSV100917583	Adenocarcinoma (prostate)
c.421T > G	p.Y108D/p.Y141D	not available	COSMIC: COSV100917488	Adenocarcinoma (rectum)
c.422A > G	p.Y108C/p.Y141C	not available	dbSNP: rs1665635198	ClinVar associated phenotypes: CI deficiency
c.589A > G	p.M164V/p.M197V	GnomAD: 0.000008 (2/250266)	dbSNP: rs771341018	
		ExAC: 0.000008 (1/120184)		
c.591G > A	p.M164I/p.M197I	ALFA: 0.00000 (0/10680)	dbSNP: rs763812241	
		ALSPAC: 0.0000 (0/3854)		
		TWINSUK: 0.0003 (1/3708)		
c.601T > G	p.F168V/p.F201V	not available	COSMIC: COSV100917478	endometrioid carcinoma
***NDUFS7***				
c.239G > T	p.W46L/p.W80L	not available	COSMIC: COSV99282674	malignant melanoma
c.256C > G	p.L52V/p.L86V	not available	dbSNP: rs758518373	
c.268G > T	p.A56S/p.A90S	not available	COSMIC: COSV52041570	ductal carcinoma (pancreas)
c.268G > A	p.A56T/p.A90T	not available	dbSNP: rs781725082	
c.268G > C	p.A56P/p.A90P	ExAC: 0.000008 (1/117774)	dbSNP: rs781725082	
c.269C > A	p.A56D/p.A90D	not available	COSMIC: COSV52039157	transitional cell carcinoma (bladder)
c.269C > T	p.A56V/p.A90V	not available	dbSNP: rs1226932595	
c.289G > C	p.A63P/p.A97P	not available	COSMIC: COSV99282661	adenocarcinoma (colon)
c.290C > G	p.A63G/p.A97G	ALFA: 0.00000 (0/11862)	dbSNP: rs1049352982	
c.291del	p.A63-/p.A97-	not available	dbSNP: rs1217004108	
c.327_328delinsGG	p.F76V/p.F110V	not available	COSMIC: COSV52040183	serous carcinoma (ovary)
c.328_329insGA	p.F76*/p.F110*	not available	dbSNP: rs1404463155	
c.339_348del	p.A81-/p.A115-	GnomAD (exome): 0.000024 (6/249810)	dbSNP: rs777504868	
		GnomAD: 0.000029 (4/140246)		
		ExAC: 0.000034 (4/119062)		
		TOPMed: 0.000026 (7/264690)		
		ALFA: 0.00000 (0/14050)

**Table 2.** MtDNA variants and amino acid position within the protein; (*) asterisk indicates the translational stop codon; (-) dash indicates frameshift variants. LHON: Leber's hereditary optic neuropathy; MELAS: mitochondrial encephalopathy, lactic acidosis and stroke-like episodes; LS: Leigh syndrome; SNHL: mitochondrial non-syndromic sensorineural hearing loss. MITOMAP: human mitochondrial genome database (https://www.mitomap.org/MITOMAP); Helix Mitochondrial database (https://www.helix.com/pages/mitochondrial-variant-database).

Nucleotide change	Aa change	MAF % (*n*. variants/total samples)	Source(s)	Notes
***MT-ND1***				
m.3368T > C	p.M21T	Mitomap: 0.035 (20/56895)	dbSNP: rs1603218920	
		Helix: 0.037 (72/195983)		
m.3376G > A	p.E24K	Mitomap: 0.000 (0/56895)	dbSNP: rs397515612	associated diseases: LHON, MELAS
m.3377A > G	p.E24G	not available	COSMIC: COSV104670687	malignant melanoma
m.3380G > A	p.R25Q	Mitomap: 0.005 (3/56895)	dbSNP: rs1603218926	endometrioid carcinoma
			COSMIC: COSV62293468	ClinVar-associated phenotypes: MELAS
m.3388C > A	p.L28M	Mitomap: 0.046 (26/56895)	dbSNP: rs387906730	ClinVar-associated phenotypes: LS, SNHL
		Helix: 0.117 (230/195983)		Haplogroups: U5a1a1i, H2a2b1a1, H2a2b1a, R8
m.3407G > A	p.R34H	Mitomap: 0.002 (1/56895)	dbSNP: rs1603218938	
m.3918G > C	p.E204D	Mitomap: 0.000 (0/56895)	dbSNP: rs28357972	
		Helix: 0.001 (1/195983)		
m.4126C > A	p.R274*	not available	dbSNP: rs28461785	

### 2.10. Statistical analyses

GraphPad v8 (GraphPad Software Inc., San Diego, CA, USA) was used to create plots and to perform statistical tests, as indicated in figure legends. Data are expressed as averages of at least three independent experiments and standard error of the mean (s.e.m.) is represented by error bars, unless explicitly indicated.

## 3. Results

### 3.1. Metformin and EVP 4593 exert CI-independent antiproliferative effect on cancer cells

To investigate the specificity of three CI targeting compounds, namely metformin, EVP 4593 and BAY 87-2243, we exploited unique cancer cell models lacking CI previously generated by knocking out the core subunit gene *NDUFS3*. In detail, we used ovarian carcinoma SKOV3 cells [45], colorectal cancer HCT116 [7] and murine melanoma B16-F10 [46] as representative of neoplasias in which metformin has shown contrasting data [23,30,58–60]. These models have been previously characterized in terms of bioenergetics and CI stability [7,45,46]*.* To investigate whether the three inhibitors may display CI-independent antiproliferative effects, growth of NDUFS3^−/−^ cells (SKOV3^−/−^, HCT116^−/−^, B16-F10^−/−^) was analysed upon treatment at concentrations earlier determined in *in vitro* experimental settings [37,41,42]. Metformin reduced cell growth of all NDUFS3^−/−^ models in most of the tested conditions (figure 1*a*; electronic supplementary material, figure S1*a*), suggesting that its antiproliferative ability is considerably mediated by CI-independent mechanisms. While HCT116^−/−^ and SKOV3^−/−^ displayed reduced cell growth with all the tested concentrations, B16-F10^−/−^ cell proliferation was reduced only at high metformin concentrations (10–20 mM) (figure 1*a*; electronic supplementary material, figure S1*a*), suggesting that the drug's CI-independent action on cell proliferation may depend on tissue or species origin. On the other hand, EVP 4593 treatment showed similar effects in all three cell lines, causing the reduction of NDUFS3^−/−^ cell growth at 20 µM in SKOV3 and B16-F10, and at 10–20 µM in HCT116 (figure 1*b*; electronic supplementary material, figure S1*b*), revealing CI-independent antiproliferative action only at high concentrations. Finally, CI-deficient cancer cell growth was unaffected by BAY 87-2243 (figure 1*c*; electronic supplementary material, figure S1*c*), indicating that under the tested conditions the compound displays no CI-independent antiproliferative effects. Hence, when comparing CI-independent effects, it appears clear that metformin and EVP 4593 decrease cancer cell growth also via mechanisms unrelated to CI (figure 1*d*), a phenomenon that may depend on the inhibitors' concentration and cancer background.

### 3.2. Apoptosis is triggered by inhibitor concentrations associated with CI-independent antiproliferative effects

Targeting CI has been proposed as a potential therapy for several cancers, particularly those commonly relying on oxidative phosphorylation (OXPHOS) for energy production. Therefore, CI-competent cells (NDUFS3^+/+^) were treated with the same concentrations of the three inhibitors. Metformin induced a dose-dependent decrease in cell proliferation, with a reduction of cell growth after 72 h of 58–90% in SKOV3, 46–77% in HCT116 and 24–62% in B16-F10 (figure 2*a*; electronic supplementary material, figure S2*a*). A similar effect was observed with EVP 4593 (2.5–20 µM), which after 72 h induced a decrease in cell proliferation of 49–62% in SKOV3, 52–74% in HCT116 and 66–84% in B16-F10 (figure 2*b*; electronic supplementary material, figure S2*b*). Conversely, BAY 87-2243 caused a modest, although significant, antiproliferative action even at the highest concentration applied (1 µM), reducing cell growth after 72 h treatment by 38%, 20% and 28%, in SKOV3, HCT116 and B16-F10, respectively (figure 2*c*; electronic supplementary material, figure S2*c*). Moreover, EVP 4593 and BAY 87-2243 induced a consistent and significantly higher block of cell proliferation in CI-competent cells compared to their CI-defective counterparts, indicating that such antiproliferative effect is mainly mediated by CI inhibition, while metformin showed this behaviour only in B16-F10 cells (figure 2*d–f*).

**Figure 3.**
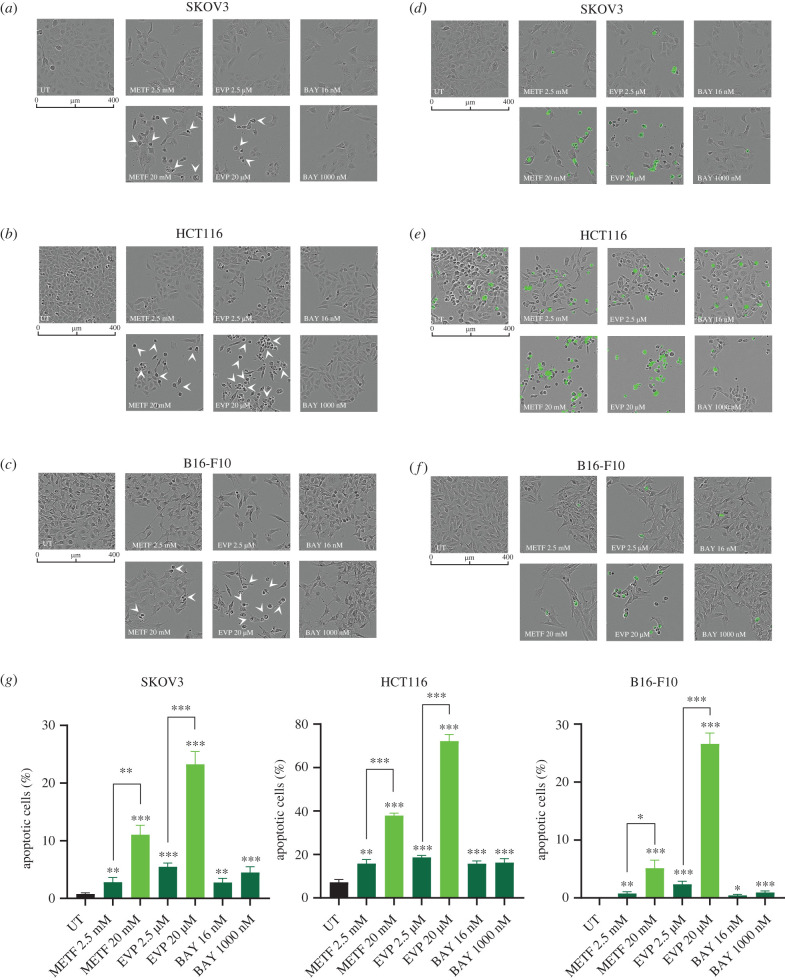
Apoptotic cell death causes CI-independent block of proliferation upon high concentrations of metformin and EVP 4593. (*a*–*c*) Representative images of SKOV3 (*a*), HCT116 (*b*) and B16-F10 (*c*) incubated for 72 h in the presence of the indicated inhibitor concentrations. Arrowheads indicate dead cells. Scale bars: 400 µm. Magnification: 10×. (*d*–*f*) Representative images of SKOV3 (*d*), HCT116 (*e*) and B16-F10 (*f*) cell lines treated with metformin, EVP 4593 and BAY 87-2243 for 72 h in the presence of caspase-3/7 activated fluorescent dye. Scale bars: 400 µm. Magnification: 10×. (*g*) Quantification of apoptotic cells at 72 h of each treatment, calculated dividing the number of positive green objects by total number of cells per field. Data (mean ± s.e.m., *n* = 3) are expressed as percentage and Student *t-*test was performed between untreated (*UT*) and treated samples. Statistical significance is specified with asterisks (*p ≤ 0.05, **p ≤ 0.01, ***p ≤ 0.001).

To understand whether the observed antiproliferative effect is a result of cell death, we next evaluated the presence of morphologically altered dying cells and caspase 3/7 activation in real time upon treatments. A small number of dying cells was observed with either the tested BAY 87-2243 concentrations or with lower doses of EVP 4593 (2.5 µM) and metformin (2.5 mM), whereas a higher percentage of cell death was found when treatments with 20 µM EVP 4593 and 20 mM metformin were carried out (figure 3*a*–*c*). Activation of caspase 3/7 in these conditions indicated the triggering of apoptosis by exposure to high concentrations of EVP 4593 and metformin (figure 3*d*–*f*). In particular, 2.5 mM metformin for 72 h induced apoptotic cell death either comparable to the untreated control (HCT116 and B16-F10) or in a relatively small portion of the cells (2.8% in SKOV3), while 20 mM metformin markedly induced apoptosis in all cell models (figure 3*g*). Similarly, a modest percentage of dead cells was observed after 72 h treatment with 2.5 µM EVP 4593, whereas 20 µM concentration of the drug caused a pronounced apoptotic cell death, ranging from 24.6% in SKOV3 to 77.6% in HCT116 (figure 3*g*). Lastly, apoptosis was observed in a very low percentage of cells treated with BAY 87-2243 (figure 3*g*), the only inhibitor for which CI-specific antiproliferative action was determined regardless of the concentrations used (figure 1*c*,*d*). Of note, apoptosis was triggered at the same metformin and EVP 4593 concentrations for which CI-independent effects were observed, suggesting that CI-specific antiproliferative action are most likely either cytostatic or related to other types of cell death.

### 3.3. Titration of inhibitors reveals that metformin-induced apoptosis does not depend on CI inhibition

**Figure 4.**
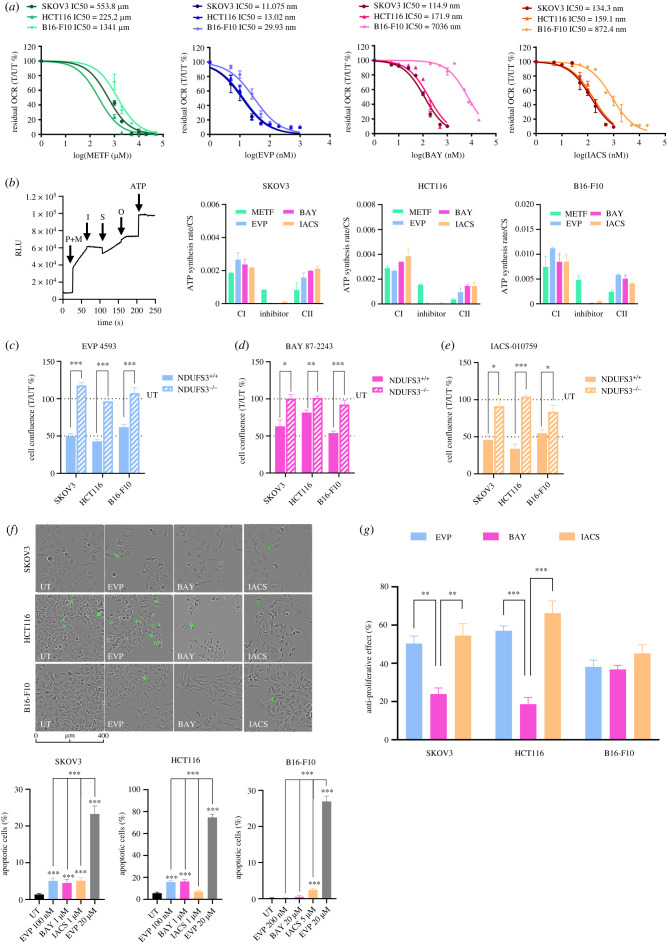
(*Overleaf*.) Optimized concentrations of EVP 4593 and BAY 87-2243 reduce cell proliferation without triggering apoptotic cell death. (*a*) Concentration-response analysis of metformin (METF), EVP 4593 (EVP), BAY 87-2243 (BAY) and IACS-010759 (IACS) on SKOV3, HCT116 and B16-F10 cell lines. Residual oxygen consumption rate (OCR) is expressed as percentage and was calculated using the last rate measurement before compound injection (*T*) to untreated (*UT*) samples. Compound concentrations were expressed as logarithm to base 10 (Log). Data were shown as mean ± s.e.m. (*n* = 2). DMSO was used as a control for EVP 4593, BAY 87-2243 and IACS-010759 untreated (*UT*) samples. For metformin titration, cells were pre-incubated with the compound for 24 h before OCR measurement. (*b*) CI- and CII-driven ATP synthesis rates in permeabilized cells. A general experiment scheme is shown as an example of the assay. Injections are indicated by arrows and correspond to CI-driven ATP synthesis using pyruvate and malate as substrates (*P* + *M*), followed by inhibitors injection (*I*), CII-driven ATP synthesis using succinate (*S*) as substrate, ATP synthase inhibition by oligomycin (*O*) and lastly, internal standard (ATP). Permeabilized SKOV3, HCT116 and B16-F10 cells were treated with metformin (50 mM), EVP 4593 (100 nM for SKOV3 and HCT116, 200 nM for B16-F10), BAY 87-2243 (1 µM for SKOV3 and HCT116, 20 µM for B16-F10) and IACS-010759 (1 µM for SKOV3 and HCT116, 5 µM for B16-F10). Data are normalized on citrate synthase (CS) activity and expressed as mean ± s.e.m. (*n* ≥ 2). Data of CI-driven ATP synthesis rate are reported as ‘CI', those after the treatment above mentioned inhibitors are indicated as ‘Inhibitor' and those regarding CII-driven are labelled as ‘CII'. (*c–e*) Proliferation of NDUFS3^+/+^ and NDUFS3^−/−^ cell lines under treatment with optimized concentrations of (*c*) EVP 4593 (100 nM for SKOV3 and HCT116, and 200 nM for B16-F10), (*d*) BAY 87-2243 (1 µM for SKOV3 and HCT116, and 20 µM for B16-F10) and (*e*) IACS-010759 (1 µM for SKOV3 and HCT116, and 5 µM for B16-F10). Data are presented as cell confluence ratio of treated (*T*) to untreated (*UT*) control cells after 72 h of incubation. Cell confluence was previously normalized on time 0 (*T*0). Student *t-*test was performed between NDUFS3^+/+^ and NDUFS3^−/−^ cells, for each cell line and treatment condition. Data are mean ± s.e.m. (*n* ≥ 4) and statistical significance is specified with asterisks (*p ≤ 0.05, **p ≤ 0.01, ***p ≤ 0.001). (*f*) Representative images of SKOV3, HCT116 and B16-F10 cell lines treated with the optimized concentrations of EVP 4593, BAY 87-2243 and IACS-010759 for 72 h, in presence of caspase-3/7 activated fluorescent dye. Scale bars: 400 µm. Magnification: 10×. Cell confluence and green fluorescence were analysed using Incucyte Live-imaging System. Quantification of apoptotic cells expressed as percentage in each treatment was calculated dividing the number of positive green objects by total number of cells per field. Cells treated with 20 µM EVP 4593 (EVP) were used as positive controls. Data are mean ± s.e.m. (*n* = 3) and Student *t-*test was performed between untreated (*UT*) and treated samples. Statistical significance is specified with asterisks (*p ≤ 0.05, **p ≤ 0.01, ***p ≤ 0.001). (*g*) Anti-proliferative effect for EVP 4593, BAY 87-2243 and IACS-010759 in SKOV3 and HCT116 (100 nM, 1 µM and 1 µM, respectively) and B16-F10 (200 nM, 20 µM and 5 µM respectively) cell lines under 72 h of treatment. Data are expressed as [100% (*UT*) – cell confluence ratio (*T*/*UT* %)]. Data are mean ± s.e.m. (*n* ≥ 3) and Student *t-*test was used to evaluate significant differences in anti-proliferative effect among the three CI inhibitors. Statistical significance is specified with asterisks (*p ≤ 0.05, **p ≤ 0.01, ***p ≤ 0.001).

The apoptotic cell death observed upon treatment with high doses of the compounds may derive from overdosage or from their inhibitory activity on molecular targets other than CI, as highlighted by the reduced cell proliferation in CI-defective cells. Hence, to identify the lowest concentrations of the three inhibitors that completely abolish CI-dependent respiration, we measured oxygen consumption rate (OCR) upon titration in CI-competent cells and validated our data using the potent and specific CI inhibitor IACS-010759 as a reference compound [9]. Independently from the cell model, acute injection of metformin (1 mM–50 mM) marginally affected OCR, inducing a 25–35% decrease of basal respiration only at the highest concentration (electronic supplementary material, figure S3*a*). This is due to the fact that metformin is a hydrophilic compound requiring internalization mediated by organic cation transporters (OCTs) [61]. Indeed, pre-incubation with increasing concentrations of metformin for 24 h was able to reduce both basal and maximal OCR (electronic supplementary material, figure S3*b*). Basal OCR was abolished by 2 mM metformin in HCT116 and SKOV3 cells and 10 mM in B16-F10 cells (electronic supplementary material, figure S3*a*). However, at these concentrations maximal respiration was still detectable, while it was abolished only when cells were pre-incubated with 50 mM metformin (electronic supplementary material, figure S3*a*). Interestingly, the concentrations commonly used in the literature (10 mM and 20 mM) were unable to completely suppress maximal mitochondrial respiration, while still being able to trigger apoptotic cell death (figure 3*g*), indicating that the latter phenotype is probably largely caused by a CI-independent activity of the inhibitor. On the other hand, EVP 4593 titration revealed that 100 nM was the lowest concentration able to fully curb mitochondrial respiration in SKOV3 and HCT116, while 200 nM was required in B16-F10 (electronic supplementary material, figure S3*c*). Hence, the apoptotic cell death observed with high concentrations of EVP 4593 was probably due to an overdosage. Lastly, we identified 1 µM as the lowest concentration of BAY 87-2243 that completely inhibited mitochondrial respiration in HCT116 and SKOV3 cells, while B16-F10 were less sensitive to this inhibitor, requiring 20 µM to suppress maximal OCR (electronic supplementary material, figure S3*d*). Similar to BAY 87-2243, the reference compound IACS-010759 abolished OCR at the concentration of 1 µM in SKOV3 and HCT116 and of 5 µM in B16-F10 (electronic supplementary material, figure S3*e*). Interestingly, concentrations that effectively inhibited mitochondrial respiration varied greatly, with IC50 ranging from 225 to 1341 µM for metformin, from 0.12 to 7 µM for BAY 87-2243 and from 11 to 30 nM for EVP 4593, with the IC50 for the reference compound IACS-010759 ranging from 134 nM to 872 nM (figure 4*a*). To further validate our results, we determined the ability of permeabilized cells to synthetize ATP specifically via CI, or via CII, or upon inhibition of CI using the three compounds, as well as IACS-010759. Similar to the reference compound, optimized concentrations of EVP 5493 and BAY 87-2243 were able to strongly inhibit CI-driven ATP synthesis in all the investigated cell models, while cells treated with 50 mM metformin still retained most of their CI-driven ATP synthesis with a percentage of inhibition ranging from 44% to 61% (figure 4*b*). Moreover, while cells incubated with optimized concentrations of EVP 5493, BAY 87-2293 and IACS-010759 showed a similar CII-driven ATP synthesis, this was reduced in presence of metformin (figure 4*b*), indicating that this molecule may interfere also with CII activity, as previously reported [35].

Next, we aimed to identify the most specific CI inhibitor with efficient antiproliferative activity. Since metformin resulted the least specific and less effective CI inhibitor and considering that the dosage required for the abolishment of maximal respiration in our models was associated with CI-independent antiproliferative mechanisms, we excluded the drug from further investigations. Then, we determined cell growth upon treatment with optimized concentrations of EVP 4593 (100 nM for SKOV3 and HCT116, and 200 nM for B16-F10) and BAY 87-2243 (20 µM for B16-F10), with the aim to determine whether they maintained an antiproliferative action. At these newly defined concentrations, the two investigated inhibitors, as well as IACS-010759, reduced cell proliferation of NDUFS3^+/+^ cells only, without affecting their CI-defective isogenic counterparts (figure 4*c*–*e* and electronic supplementary material, figure S4). Moreover, the massive apoptotic cell death observed in cells treated with 20 µM EVP 4593 was not detected with the optimized concentration (figure 4*f*), supporting the idea that specific CI inhibition may not induce cytotoxicity, but mainly slows down cell growth. Finally, the comparison between antiproliferative effect revealed that IACS-010759 and EVP 4593 are more effective than BAY 87-2243 in human SKOV3 and HCT116 cells, while in murine B16-F10 cells the three molecules are comparable (figure 4*g*).

Overall, these data show that metformin is not a CI specific inhibitor and that it prevents cancer cells proliferation by hitting also other intracellular targets, while EVP 4593 and BAY 87-2243 resulted highly specific, but effective at different concentrations.

### 3.4. Molecular docking predicts differential binding for EVP 4593 and BAY 87-2243 to the Q-site of CI

**Figure 5.**
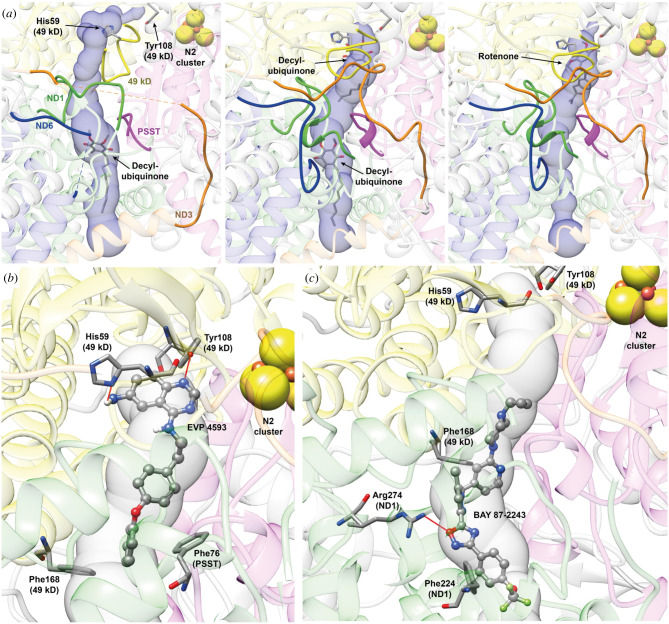
Molecular docking reveals different binding sites for EVP 4593 and BAY 87-2243 in ovine respiratory CI. (*a*) Q-site channel departing from Tyr108 (49 kDa) calculated using the CI structure in the initial step of the catalytic cycle (PDB ID 6ZKI, left panel), in the close conformation corresponding to the quinone reduction step (PDB ID 6ZKC, central panel) and in the rotenone inhibited structure (PDB ID 6ZKK, right panel). Protein backbones are reported in transparent cartoons coloured in grey, except for the 49 kDa, PSST, ND1, ND3 and ND6 subunits that are in yellow, purple, green, orange and blue, respectively. The loops cited by Kampjut *et al*. [44] are in solid ribbons. Decyl-ubiquinone and rotenone found in the structures are reported in sticks coloured according to the atom type, as well as His59 (49 kDa) and Tyr108 (49 kDa). The N2 [4Fe4S] cluster is shown as spheres coloured according to the atom type. The Q-site channel is reported in transparent light blue, to show the position of the ligand in the cavity. (*b*,*c*) Detail of the best docking poses for EVP 4593 (*b*) and BAY 87-2243 (*c*) calculated on the CI structure in the close conformation corresponding to the quinone reduction step (PDB ID 6ZKC). Inhibitors are shown in ball-and-stick coloured according to the atom type, while CI residues forming specific interaction with the ligands are in stick coloured accordingly to atom type. H-bonds are shown using red lines.

In order to understand whether the different efficiency of EVP 4593 and BAY 87-2243 may depend on the structural determinants of their interactions in the Q-site of CI, we performed a docking analysis exploiting the recently released high resolution structures of ovine CI [44]. We decided to focus on the Q-site, considering that, as several other inhibitors, EVP 4593 and BAY 87-2243 are supposed to bind into the worm like channel formed by ovine subunits PSST (NDUFS7 in human CI), 49 kDa (NDUFS2 in human CI) and ND1 [36,40,44,62–65]. The analysis with MOLEonline showed that the Q-site assumes different shapes depending on the conformation of the CI itself and on the bound molecule(s) (figure 5*a*). Indeed, in the case of the quinone reduction step (6ZKC, figure 5*a* central panel), the channel is wide open in both the shallow and the deep sites. On the other hand, in the case of the initial step of the catalytic cycle (6ZKI, figure 5*a*, left panel) and in the rotenone inhibited form (6ZKK, figure 5*a*, right panel), the channel narrows considerably at the deep and at the shallow site, respectively. Moreover, in the initial step of the catalytic cycle (6ZKI), the deep site is no longer in contact with Tyr108 (49 kDa), that is the residue supposed to pass the electron received from the N2 [Fe4S4] cluster located at the end of the iron–sulfur cluster chain starting on the top of the CI side arm.

Based on the results of the redocking stage and the conformations adopted by the Q-site channel (electronic supplementary material, figure S5*a*), we decided to use only the structure of CI in the quinone reduction step (6ZKC) for docking and characterization of the intermolecular interactions of EVP 4593 and BAY 87-2243. Considering the different chemical groups composing the two inhibitors, a different behaviour in the interaction with the Q-site was expected. Indeed, the EVP 4593 best docking pose is located in the deep site of the channel, similar to that observed for rotenone and decyl-ubiquinone (figure 5*b* and electronic supplementary material, figure S5*b*,*c*). The binding pose is stabilized by the formation of one H-bond between EVP 4593 quinazoline nitrogen N1 and Tyr108 (49 kDa) O*ζ*. Moreover, the EVP 4593 amino group bound to the quinazoline moiety is very close to His59 (49 kDa) N*δ*1 in a geometry unsuitable for hydrogen bond formation. The docking program does not allow for the optimization of the geometry of the protein, but the formation of an additional H-bond may be hypothesized (figure 5*b*). Finally, EVP 4593 binding pose is stabilized by π-stacking interactions between Phe168 (49 kDa) and Phe76 (PSST) and the EVP 4593 oxydibenzene moiety at the opposite side of the quinazoline group. Conversely, BAY 87-2243 best binding pose is found in the central part of the Q-site channel, in direct contact with the shallow site together with a large portion of the region between the latter and the deep site (figure 5*c* and electronic supplementary material, figure S5*b*,*c*). BAY 87-2243 inserts into the Q-site using its cyclopropyl terminal, localized in the proximity of Thr49 (PSST) and Met60 (PSST), while the trifluoromethyl end is located in the vicinity of the aliphatic part of Asp51 (ND1) side chain and Phe224 (ND1). The binding pose is stabilized by a H-bond between the oxygen atom of BAY 87-2243 oxadiazole moiety and the Arg274 (ND1) N*ω*2 atom, together with a perpendicular π-stacking interaction between the BAY 87-2243 pyridinic ring and Phe168 (49 kDa) and a parallel π-stacking between the phenyl moiety bound to the oxadiazole moiety of the inhibitor and the already mentioned Phe224 (ND1). However, the largest part of the intermolecular interactions between BAY 87-2243 and the Q-site channel are van der Waals contacts and the stabilization of the binding pose appears to be mainly due to shape complementarity. This result is in agreement with the binding pose of a similar compound (IACS-2858) recently co-solved with murine CI [66], where BAY 87-2243 and IACS-010759 have been superimposed and minimized on IACS-2858 structure.

Overall, these data suggest that the higher efficacy of EVP 4593 compared to BAY 87-2243 may reside in their different interaction mode with the Q-site of respiratory CI, where EVP 4593 forms a tighter network of interactions at the level of the deep site, resembling the interactions made by the substrate mimetic decyl-ubiquinone or the classic specific CI inhibitor rotenone.

### 3.5. Variants mapping within the inhibitor-binding Q-site that may affect inhibitors efficiency occur at low frequency in the human population

Interestingly, EVP 4593 showed a similar IC50 range in all the investigated cell lines (figure 4*b*), while BAY 87-2243 was 50-fold less efficient in murine B16-F10 cells (IC50 = 7 µM) compared to human models (IC50 = 115–171 nM) (figure 4*a*). We hypothesized such difference may result from changes in the residues interacting with BAY 87-2243 between human and murine CI. Hence, we investigated the conservation of the amino acids predicted to bind EVP 4593 and BAY 87-2243 in the deep and shallow Q-sites, respectively (electronic supplementary material, table S1). The deep Q-site presented 8/10 conserved amino acids predicted to interact with EVP 4593 and all of them are identical in human, mouse and ovine reference sequences, in agreement with the similar IC50 found in the different cell models. The shallow Q-site showed 13/16 conserved amino acids predicted to interact with BAY 87-2243. All residues are identical in human, mouse and ovine reference sequences, with the only remarkable exception of Thr21(ND1), which is found in mouse and ovine sequences, but corresponds to a methionine in human. This amino acid was conserved in primates, clearly indicating that it has been recently acquired during evolution and may concur to the stabilization of BAY 87-2243 in the shallow Q-site, possibly due to its higher hydrophobicity and propensity to form van der Waals interactions.

We also considered that the amino acids predicted to interact with EVP 4593 and BAY 87-2243 may be subjected to physiological genetic variation and/or somatic mutational events, whose identification could potentially steer therapeutic decisions within the context of personalized therapy. Therefore, we performed a search for variants in the three genes, namely *MT-ND1*, *NDUFS2* and *NDUFS7*, with the aim to gauge whether genetic changes possibly affecting the molecular interactions that EVP 4593 and BAY 87-2243 form in the Q-site may be frequent events. We extracted six variants for *NDUFS2* and 13 for *NDUFS7* (table 1), while *MT-ND1* analysis identified a total of eight variants (table 2). Where available, MAFs of the germline nuclear variants were less than 1% with the respect to the general population; *MT-ND1* variants had frequencies less than 0.1%, and COSMIC variants were also rare somatic events.

These results suggest that EVP 4593 and BAY 87-2243 may be potentially used on a large scale, as their binding sites do not suffer from a high genetic variability within the population or at a somatic level.

## 4. Discussion

In this work, we compared the antiproliferative effects of three CI inhibitors which have been proposed as anti-cancer agents, namely metformin, BAY 87-2243 and EVP 4593, in different cancer cell models and investigated the interactions of those specific for CI at the atomic levels in the Q binding site. To this aim, we exploited unique cell models in which the genetic ablation of *NDUFS3* induces the almost complete disassembly of CI (residual activity of less than 10%) with a selective degradation of the structural modules N, Q and proximal P [7,46], thus destroying the Q-site in which most CI inhibitors bind. In these cells, we demonstrated that the antiproliferative effects of metformin and EVP 4593 at concentrations commonly used in *in vitro* experiments [4,33,41] are mainly due to CI-independent mechanisms and are associated with triggering of apoptosis. Further, in syngeneic CI-competent cancer models (NDUFS3^+/+^) we found that metformin treatment induces apoptosis at concentrations at which CI is not fully inhibited, indicating that the antiproliferative effects of this drug result also from the inhibition of other targets. These data are in line with recent studies in which metformin has been shown to curb other metabolic pathways such as gluconeogenesis from lactate and glycerol [32] and fatty acid oxidation [67], or to target other mitochondrial enzymes, such as F_1_F_o_ ATP synthase [33] and glycerophosphate dehydrogenase (mGPDH) [32,34,35]. Moreover, the recent release of mammalian CI structures binding IM1761092, a more complex and hydrophobic phenformin derivative that has a higher inhibitory activity on CI, revealed that biguanides are able to bind at least three sites in deactive CI explaining the non-competitive behaviour of these molecules [68], but also raising questions regarding their specificity and potency. Importantly, our results can contribute to explain the conflicting data obtained in clinical trials on the use of metformin as an anti-cancer drug for different neoplasias [20–23,27,29,30,60], which may express diverse levels of additional yet unknown molecular targets.

On the contrary, 20 µM EVP 4593 stimulates apoptosis most likely because of an overdosage of the compound. In particular, in our models EVP 4593 is able to completely suppress mitochondrial respiration in a concentration range of 100–200 nM, thus 100–200 times lower than the doses usually employed in literature *in vitro* [40,41]. Indeed, when appropriate amounts of EVP 4593 were used, the compound was highly selective for CI and did not trigger massive apoptosis. Hence, the cytotoxic effect shown by EVP 4593 overdosage may be related to other molecular targets than CI. In this frame, it is important to note that this compound has only recently been recognized as a CI-targeting molecule [40] and was initially considered as a high-affinity antagonist of the NF-κB pathway [69,70]. Whether such inhibition secondarily derives from the block of CI activity or is due to a direct targeting of NF-κB remains to be elucidated. For human cancer cells, SKOV3 and HCT116, the highest concentration of BAY 87-2243 used in the literature (1 µM) was the minimal amount suppressing mitochondrial respiration, thus already optimal for further experiments. Hence, both EVP 4593 and BAY 87-2243 resulted highly specific CI inhibitors and slowed down cancer cell proliferation without being generally cytotoxic when used at appropriate concentrations, in accordance with previous reports [36,37,71]. This supports their use as adjuvant agents that may open a therapeutic window for other interventions, although the molecular mechanism(s) reducing cell proliferation remains to be elucidated. In this context, it is important to note that, with the aim to define optimal concentrations, the CI inhibitor specificity should be evaluated also in the preclinical *in vivo* testing, possibly by comparing the antiproliferative effect between CI competent and CI knockout cancer models, in analogy to the experiments performed in this work. Our data pave the way for relevant considerations on the potential side effects derived by the use of inhibitors of such a ubiquitous and pivotal enzyme as CI in cancer treatment. Of note, inherited genetic defects of structural subunits or assembly factors of CI are etiological of mitochondriopathies, ranging from severe neuromuscular disorders such as Leigh syndrome, mitochondrial encephalopathy, lactic acidosis and stroke-like episodes (MELAS) syndrome and hypertrophic cardiomyopathies to mitochondrially inherited or recessive forms of optic atrophy (reviewed in [72]). Indeed, targeting CI by some classic inhibitors (i.e. rotenone, MPP^+^ or paraquat) has been demonstrated to promote the occurrence of neurodegenerative disorders such as Parkinson disease [73]. Moreover, such inhibitors have been reported to stimulate reactive oxygen species production, which are well-known modulator of tumour progression and aggressiveness [74]. With respect to this, we never observed an increase of cell proliferation in all the investigated models and for all tested conditions, indicating that reactive oxygen species production may not be relevant in the mechanism of action of EVP 4593, BAY 87-2243 and metformin at the concentrations that fully inhibit CI. Hence, a well-tolerated and precise dosage of CI-specific inhibitors is particularly advisable to avoid detrimental side effects which may involve the nervous system, as well as cardiac and skeletal muscles, or increase tumour progression and aggressiveness.

Noteworthy, EVP 4593 and BAY 87-2243 completely suppress mitochondrial respiration at different concentrations in human cells derived from ovarian and colorectal cancer, unlike in murine melanoma cells. Although tumour type, mutational burden or metabolic peculiarities of cells may not be ruled out in explaining such difference, modifications at the level of the Q-site among species may represent the most plausible determinant of the phenomenon. Indeed, the docking calculations predict EVP 4593 to bind the deep Q-site, similarly to classic CI inhibitors rotenone and piericidin A, while the shallow Q-site should harbour BAY 87-2243, whose binding is mainly due to van der Waals interactions and size complementarity, in a region characterized by hydrophilic residues [75]. Whether such difference in the binding site may affect the ability of these inhibitors to compete with quinone remains an open question. Lastly, it is important to note that substitutions of single critical amino acids may affect the IC50 of these inhibitors [66], as in the case of here shown Thr21 found in murine and ovine ND1 which is substituted by a methionine in human cells, possibly rendering the latter more sensitive to BAY 87-2243. However, the analysis of germline or somatic variants inducing amino acids substitutions in the shallow and deep Q-site showed that genetic variability is very low at these regions, supporting the use of EVP 4593 and BAY 87-2243 on a large set of oncological patients.

To conclude, this is the first comparative work on the effects of three CI inhibitors proposed as potential anti-cancer molecules, namely metformin, EVP 4593 and BAY 87-2243. We here highlight the importance of detailed investigations on the properties of these compounds, in particular exploiting appropriate cell models, such as CI-defective cancer cells generated in different backgrounds in order to optimize effective doses avoiding the inhibition of off-target molecular players. Moreover, we show that functional analyses combined with molecular docking provide structural insights on the mechanism of action of CI inhibitors that eventually may help the drug design process. Given the accumulation of somatic mtDNA mutations in human cancers, which may affect the specificity of such inhibitors, this combined approach could be helpful in a personalized medicine perspective.

## Data Availability

The datasets analysed during the current study are available from the corresponding authors on reasonable request. Materials are available upon request. Data are provided as compressed folders and available at this link https://site.unibo.it/mitobb/it/downloads Supplementary material is available online [76].
